# Photocatalytic Degradation Studies of Organic Dyes over Novel Cu/Ni Loaded Reduced Graphene Oxide Hybrid Nanocomposite: Adsorption, Kinetics and Thermodynamic Studies

**DOI:** 10.3390/molecules28186474

**Published:** 2023-09-06

**Authors:** Amina Kanwal, Tayyaba Shahzadi, Tauheeda Riaz, Maria Zaib, Safia Khan, Mohamed A. Habila, Mika Sillanpaa

**Affiliations:** 1 Department of Chemistry, Government College Women University Sialkot, Sialkot 51310, Pakistan; aminakanwal11@yahoo.com (A.K.);; 2 Department of Chemistry, University of Jhang, Jhang 35200, Pakistan; 3 Shandong Technology Centre of Nanodevices and Integration, School of Microelectronics, Shandong University, Jinan 250101, China; safiakhan@chem.qau.edu.pk; 4 Department of Chemistry, College of Science, King Saud University, P.O. Box 2455, Riyadh 11451, Saudi Arabia; aa9868591@gmail.com; 4 Department of Biological and Chemical Engineering, Aarhus University, Nørrebrogade 44, 8000 Aarhus, Denmark; timsheet123@gmail.com

**Keywords:** Cu/Ni/rGO nanocomposites, rhodamine B, alizarine R, kinetics, adsorption isotherms, thermodynamic, regeneration, scavenging

## Abstract

Nowadays, for environmental remediation, photocatalytic process involving graphene-based semiconductors is considered a very promising oxidation process for water treatment. In the present study, nanocomposite (Cu/Ni/rGO) has been synthesized by *Dypsis lutescens* leaf extract. Characterization of the sample was carried out by UV-visible spectroscopy, scanning electron microscopy (SEM), energy dispersive X-ray (EDX) analysis, Fourier transform infrared spectroscopy (FTIR), and X-ray diffraction (XRD). Different parameters like contact time, nanocatalyst amount, dye concentration, effect of temperature. and pH factor were optimized to examine the maximum removal efficiency for dyes rhodamine B and alizarine R with and without visible light source. In both cases, i.e., with or without light, maximum removal was observed at 20 mg of nanocatalyst for 5 ppm concentration of both dyes at 45 °C temperature and pH 10 for rhodamine B and pH 4 for alizarine R, respectively with a 20 min contact time. Maximum removal of dyes 93% rhodamine B and 91% alizarine R were observed under a tungsten lamp as compared to without a tungsten lamp, i.e., 78% of RhB and 75% of AR from mixture solution of these dyes. To assess the rate of reaction, spontaneity, and nature of reaction thermodynamics, kinetics and adsorption isotherms were studied. Thermodynamic values indicated that both dyes depicted endothermic and spontaneous degradation processes. Isotherm data fitted best to a Freundlich isotherm, while results of kinetic studies of both dyes followed the pseudo 2nd order kinetic equation. In the end, scavenging radical studies concluded that hydroxyl radicals were the main active specie involved in the photocatalytic degradation process, and regeneration experiments resulted that Cu/Ni/rGO nanocomposites were re-utilized for about four times.

## 1. Introduction

Discharging industries effluent like paper, rubber, fabric, and leather is contributing towards massive amounts of organic (dyes, pesticides, PCBs, VOCs) and inorganic (nonmetals, heavy metals, radionuclides) contaminants [1]. Most of these pollutants are the colored substances, predominantly dyes. In textile industries, 10^3^ tons dyes per year are being consumed, and 10–15% of this amount is released into the effluent. Presence of dyes can be hazardous to aquatic life and humans as well. In case of human beings, dyes can act as carcinogenic, mutagenic, or allergenic agent [2]. In aquatic systems, dyes prevent the sunlight from penetrating and retard the photosynthesis process. Consequently, prior to disposal of industrial effluent, it is necessary to eliminate or minimize the concentration of dyes to a permissible concentration [3].

It is difficult for dyes to biodegrade because of their complex molecular structures [4,5,6]. Different techniques have been utilized for the treatment of effluent with dyes like ion exchange, biodegradation, coagulation, chemical precipitation, membrane filtration, flocculation, adsorption, and so on [7,8,9,10]. Due to poor working efficiencies and extortionate prices of all other methodologies, adsorption has been considered as economical and versatile technique. According to literature reviews, different kinds of adsorbent materials have been utilized for industrial waste water treatment. Among these materials, nanocomposites were also considered as an efficient material due to their physical, thermal, and chemical properties. They show higher surface area, diffusion rate, and porosity [11,12].

Porous material like graphene is considered the best support for metallic nanoparticles and have excellent properties like large surface area, lower density, and volume of cavities [13]. Graphene is an excellent 2D, one atom thick layer material, and it is the thinnest material in the world. Despite having excellent properties, graphene itself cannot be used at large scale for light absorption experiments. Therefore, 3D structures were formed when they are combined with nanoparticles [14,15]. Different metals, metal oxides, and semiconductor materials can be added to graphene to attain a composite format. Recently, metal reduced graphene oxide (GO) composites have attained more consideration due to their extraordinary features. With the presence of inorganic elements, graphene can constrain the aggregation of graphene sheets and maintain the high pore volume and surface area, which is considered to be a pre-eminent factor in pollutant adsorption and removal studies [16,17,18].

In this study, graphene-based metal nanocomposites (Cu/Ni/rGO) have been reported. Leaves extract of golden cane palm (*Dypsis lutescens*) were used for the preparation of the nanocomposite. Major phytochemical constituents in *D. lutescens* leaf extract are flavonoids (apeginen, vicenin, vitexin, perchafuroside, violanthin, orientin, isoorientin, luteolin), and phenolic acids (*p*-hydroxybenzoic acid, gallic acid) [19,20]. These phytochemicals act as reducing and capping agents in the preparation of the nanocatalyst. Diverse characterization techniques like UV/Vis, FTIR, XRD, EDX, and SEM were utilized to characterize the synthesized material. Initially, various parameters were analyzed on standard solutions of dyes by using Cu/Ni/rGO material as a nanocatalyst. Then, kinetic, thermodynamic, and different isotherm models were applied to evaluate the interaction mode.

## 2. Results and Discussion

### 2.1. Characterizations

#### 2.1.1. SEM and EDX Analysis

**Figure 1:**
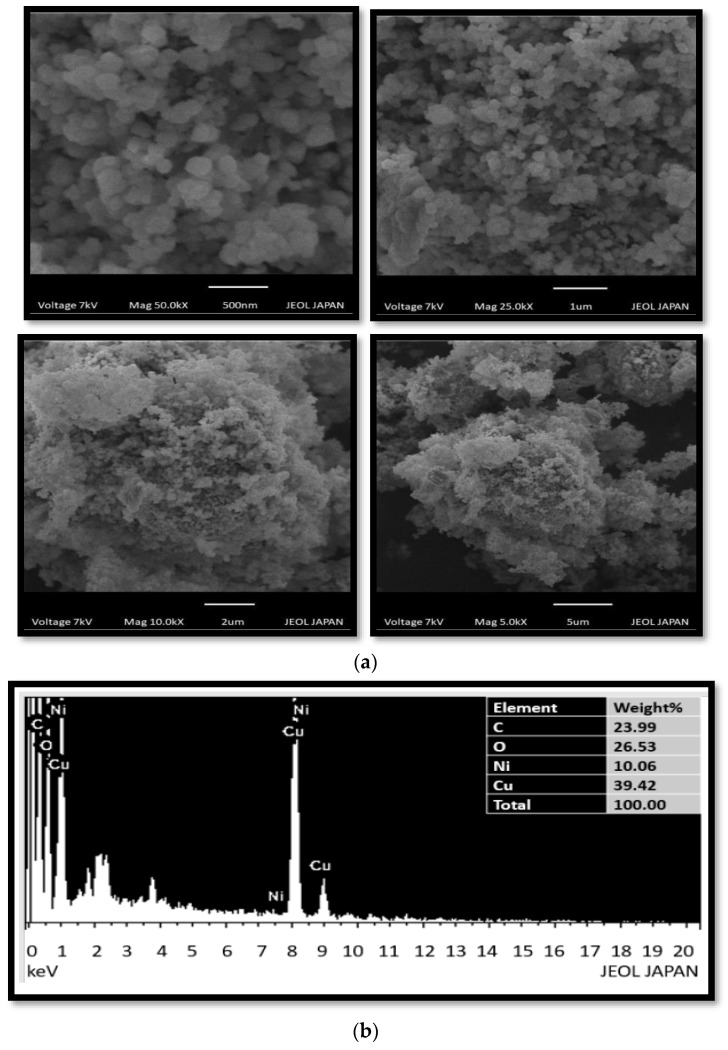
(**a**) SEM images of Cu/Ni/rGO composites at 500 nm, 1 µm, 2 µm, and 5 µm scale. (**b**) EDX spectrum of synthesized Cu/Ni/rGO composites.

To evaluate the morphology, SEM analysis was carried out at different scale level of 500 nm, 1 µm, 2 µm, and 5 µm. SEM images (Figure 1) showed the spherical shape and agglomeration of Cu/Ni/rGO composite. Agglomeration occurs because of the polymeric nature of nanoparticles, and thus clusters can be seen in the SEM results. Another reason could be the presence of phytochemicals present in leaf extract that played a major role in synthesizing and stabilizing the nanocomposites [21].

EDX analysis was used to detect the composition of synthesized nanocomposite. EDX images (Figure 1b) showed the surface of rGO was occupied by copper and Ni nanoparticles. The presence of C, O, Ni, and Cu peaks confirmed the successful synthesis of Cu/Ni/rGO composite. It also showed that all predictable elements were available in the synthesized nanocomposite [22].

#### 2.1.2. UV/Visible Spectroscopy

**Figure 2:**
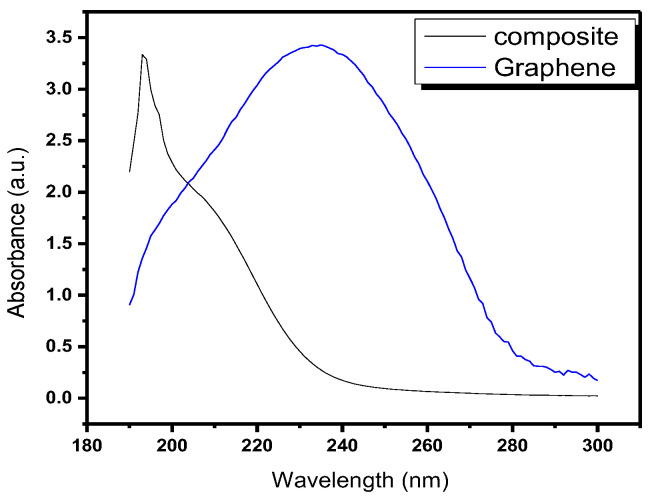
UV-visible spectrum of GO and Cu/Ni/rGO composites.

The UV-vis spectrum of nanocomposite is depicted in Figure 2. Broad adsorption peak within the wavelength range of 220–280 nm can be seen in case of GO. Shifting of the peak towards 180–200 nm confirmed the synthesis of Cu/Ni/rGO composite. Intensity and presence of a single peak indicated the high yield and purity of the nanocomposite [23].

#### 2.1.3. FTIR

**Figure 3:**
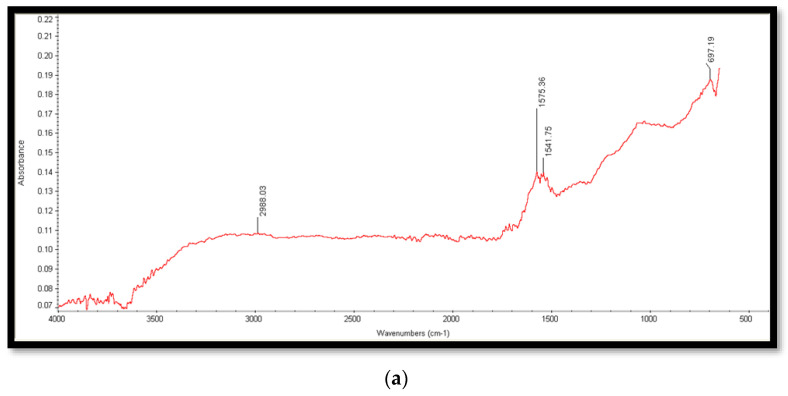

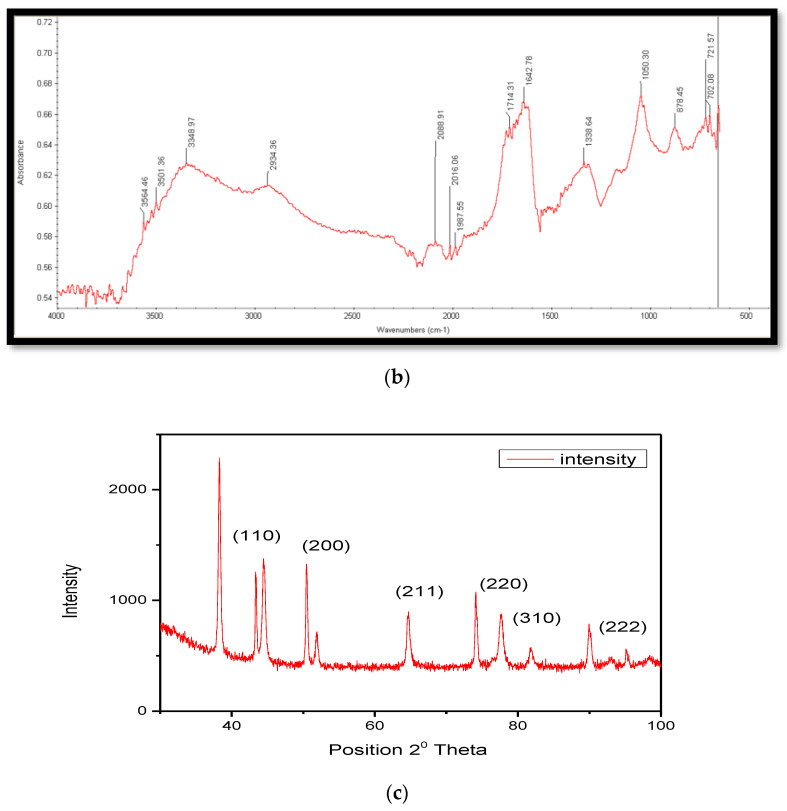
FTIR spectrum of (**a**) GO and (**b**) Cu/Ni/rGO composites, (**c**) XRD spectrum of Cu/Ni/rGO composites.

In the FTIR spectrum of the GO (Figure 3a) peak at 697 cm^−1^ attributed to C–O stretching vibrations, two peaks at 1541.75 cm^−1^ and 1575.36 cm^−1^ are due to the stretching vibrations of C=O, and one peak at 2988.03 cm^−1^ is attributed to OH stretching vibrations. The presence of functional groups containing oxygen proved that the graphite was oxidized to GO and presence of OH groups agreeing GO to quickly establish hydrogen bonds with H_2_O, giving it a hydrophilic appearance [24,25].

The FTIR spectrum of Cu/Ni/rGO (Figure 3b) composites showed different peaks at 3554 cm^−1^, 3501 cm^−1^, 334 cm^−1^, 2934 cm^−1^, 2088 cm^−1^, and 2016 cm^−1^ are due to vibrations of OH functional groups. Peaks at 1987 cm^−1^, 1714 cm^−1^, 1642 cm^−1^, and 1338 cm^−1^ are due to C=O groups. Bands at 1050 cm^−1^, 878 cm^−1^, 721 cm^−1^, and 702 cm^−1^ showed stretching and bending vibrations of C–O functional groups [26]. It is noted that after reduction, peaks of GO either vanished or appeared with considerably reduced intensities.

#### 2.1.4. XRD

Diffracted intensities were examined in the range of 0° to 120° diffracted angles (Figure 3b). In the present study, six diffracted peaks were observed at 44.39° (110), 64.58° (200), 81.72° (211), 98.15° (220), 115.26° (310), and 135.42° (222). Absences of a characteristic peak at 10° of GO indicated that a reduced form of GO hybrid composite has been formed. Intensity of peaks in the XRD pattern confirmed the crystalline nature of Cu/Ni/rGO composite. Size of composites was calculated by the Debye-Scherrer equation:

D = (kλ/β cos θ)(1)

Here, K is used as proportionality constant having 0.9, β denotes (FWHM) full width at half maximum, and λ is the wavelength of the X-ray. Calculated size was found to be 6.5 nm which was consistent with the literature [27,28]. Crystallographic parameters were also studied that confirmed the cubic nature of the crystal. Lattice parameters (a,b,c) are shown in Table 1.

#### 2.1.5. PZC (Point of Zero Charge)

**Figure 4:**
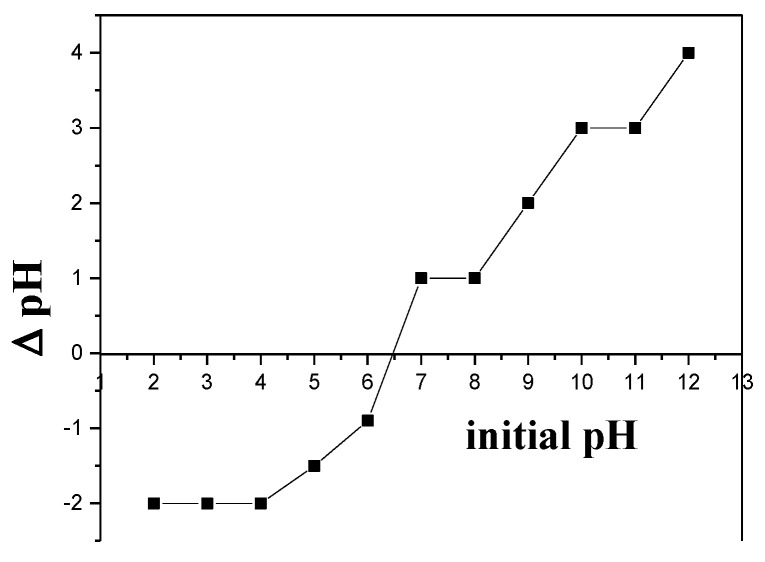
Calculated pzc value of synthesized Cu/Ni/rGO composites.

Point of zero charge is an essential property of an adsorbent and it is directly linked to the efficiency of the dye removal mechanism. In this experimental work to calculate the pzc, the salt addition method was used. The point of zero charge (pzc) of synthesized nanocomposite (Cu/Ni/rGO) was 6.5 as shown in Figure 4. When the pH of solution is higher than pzc, the surface became negatively charged, and this surface of adsorbent is more compatible for cationic dye removal from the dye mixtures, while at lower pH, the value of solution surface of nanocatalyst became positively charged which is more suitable for anionic dye removal.

### 2.2. Degradation Studies of Dyes

The dye degradation process started when a natural or artificial source of light falls on the catalyst. At the point when a catalytic material is irradiated with photons whose energy is higher or equivalent to its band hole energy, movement of an electron from the valence band (VB) to the conduction band (CB) happens with the corresponding generation of a hole in the valence band (VB).

Working in a water-based system, oxygen adsorbed on the outer layer of the catalyst acts as an electron acceptor, while the adsorbed water particles and hydroxyl anions act as electron donors, prompting the development of extremely strong oxidizing •OH radicals. Superoxide anions are produced when electrons interact with oxygen. When dye molecules adsorbed on the catalyst surface, •OH reacts to form adducts and fragments of different intermediates until the complete degradation of the dyes occurs [29].

The process is completed in the following steps.

NC + hv → NC (e^−^ + h^+^);

NC (h^+^) + H_2_O → NC + ^•^OH + H^+^;

NC (e^−^) + O_2_ → NC + O^−2^;

NC (h^+^) + dye → NC + dye^+^;

OH + dye → intermediate products → CO_2_ + H_2_O;

Here NC is indicated the nanocomposites.

#### 2.2.1. Effect of Photocatalyst Amount

**Figure 5:**
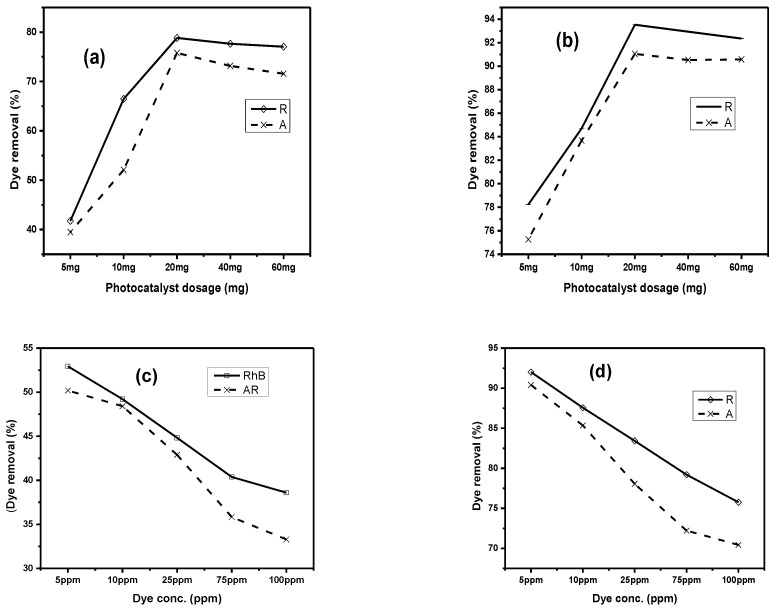

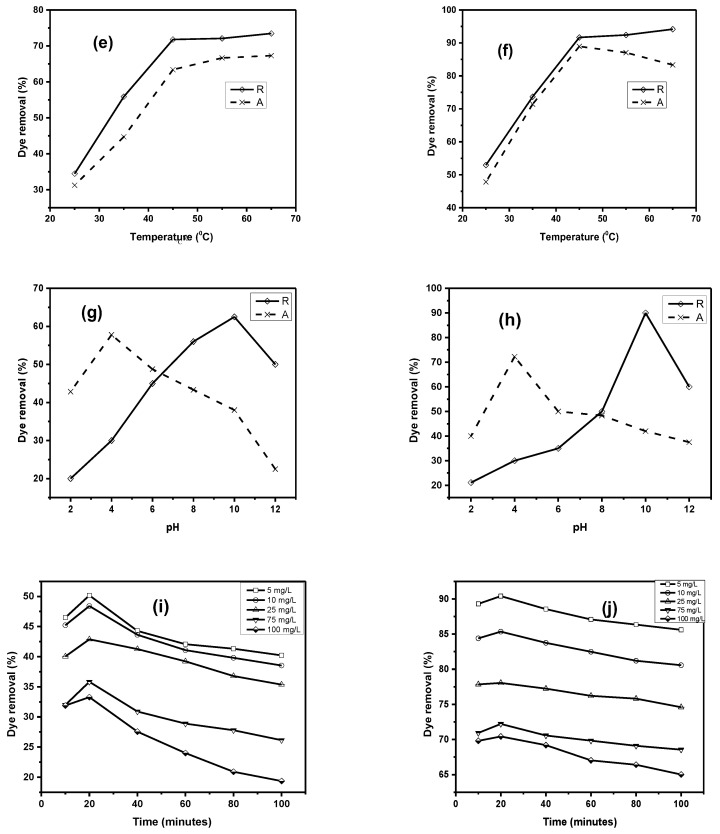
Effect of photocatalyst dosage (**a**) without tungsten lamp and (**b**) under tungsten lamp. Effect of dye concentration (**c**) without tungsten lamp and (**d**) under tungsten lamp. Effect of temperature (**e**) without tungsten lamp and (**f**) under tungsten lamp. Effect of pH (**g**) without tungsten lamp and (**h**) under tungsten lamp. Effect of time (**i**) without tungsten lamp and (**j**) under tungsten lamp.

Initial amount of photocatalyst is very important because a fixed amount of synthesized composite can degrade a limited amount of pollutant. In order to check the effect of Cu/Ni/rGO composite dosage on the binary system of dyes, a 25 mL of solution containing the same and fixed concentration (5 ppm) of two dyes (i.e., rhodamine B and alizarin R) with different photocatalyst dosage (5 mg, 10 mg, 20 mg, 40 mg, 60 mg) was kept at room temperature (25 °C) under constant stirring conditions for 2 h in the absence and presence of a visible light source as shown in Figure 5a,b. The optimal amount of photocatalyst was recorded as 20 mg for 25 mL of dye solution. Without a tungsten lamp, the % age removal of dyes was up to 78% of RhB and 75% of AR. But when the experiment was performed under a tungsten lamp, the percentage dye removal was 93% of RhB and 91% of AR. This is because the light source has facilitated the charge separation with decreased recombination rates. This leads to improved percentage values of dye removal studies. Further, it was observed that by increasing dosage amount, the percentage removal was increased up to a certain level. The reason behind the increasing percentage removal was the accessibility to more active sites of synthesized composites for the degradation of dye molecules [30,31].

#### 2.2.2. Effect of Dye Concentration

To study the impact of dye concentration, binary solutions of both dyes with different concentrations of (5 mg/L, 10 mg/L, 25 mg/L, 75 mg/L, 100 mg/L) were prepared. The optimal photocatalyst dosage, i.e., 20 mg, was added into the 25 mL binary system of dyes. After constant stirring for 2 h, it was observed that the dye removal percentage decreases with an increase in dye concentration as shown in Figure 5c,d. When the dye concentration increases, active sites of adsorbent decreases due to monolayer formation and equilibrium establishment [32]. Maximum dye removal was observed at lower dye concentration, i.e., 5 mg/L. It was 56% for rhodamine and 51% for alizarine dye in the absence of tungsten lamp. The same experiment was performed under tungsten lamp, and dye removal % was increased up to a value of 92% for rhodamine and 90% for alizarine dye.

#### 2.2.3. Effect of Temperature

Temperature is an important factor that impacts the structure of dye molecules and the interaction of dye with the photocatalyst. Therefore, at different temperatures (25 °C, 35 °C, 4 °C, 55 °C, and 65 °C), the interaction of dyes with the photocatalyst was examined as shown in Figure 5e,f. Initially, an increase in temperature up to 45 °C favors the percentage removal value of both the dyes. The reason behind this phenomenon may be owed to the increased mobility and decreased solubility of dye molecules [33]. Maximum dye removal was 71% for rhodamine and 63% for alizarine without aid of the tungsten lamp. Under a tungsten lamp, dye removal was 91% and 89% for rhodamine and alizarin dye, respectively. Rhodamin B is stable up to 195 °C temperatures and alizarin R is stable at temperatures greater than 100 °C, so these dyes will degrade at very high temperatures, which is not possible in our climatic environment. Therefore, an adsorbent like the Cu/Ni/rGO catalyst can degrade the dyes RhB and AR just at 45 °C.

#### 2.2.4. Effect of pH

The interaction of dye molecules with a nanocatalyst depends upon the surface properties of composites, and the degree of ionization could be altered due to hydrogen ion concentration present in the reaction mixture. Therefore, hydrogen ion concentration should be optimized during the degradation study. To study the impact of pH, a pH range of 2–12 was adjusted as shown in Figure 5g,h. Fixed nanocatalyst dosage (20 mg) with constant volume of binary system (25 mL) and 5 ppm dye solution was used for the whole process. Rhodamine is a cationic dye, and its attained maximum removal value of 62% at 10 pH without using tungsten lamp and 90% dye removal was examined under a tungsten lamp. At higher pH values, the surface of the nanocatalyst was negatively charged, and it attracts rhodamine dye molecules which are positively charged. Thus maximum removal occurred at higher pH values. Alizarine is an anionic dye and it shows 57% maximum removal at an acidic pH value of 4 without a tungsten lamp and 87% when the experiment was performed under a tungsten lamp. The reason behind the dye removal at a lower pH value was the interaction of the positively charged surface of nanocomposites and the negative part of the dye molecule [34].

#### 2.2.5. Effect of Contact Time

Time factor was optimized within the value of 10 to 100 min for 25 mL of a binary system with a nanocatalyst dosage of 20 mg as shown in Figure 5i,j. The experiment was performed under a tungsten lamp as well as without using a tungsten lamp. In both cases, it was observed that initial removal percentage was enhanced, but with the passage of time, the removal percentage moves towards constant values. Without the tungsten lamp, rhodamine dye removal was up to 53%, while alizarin dye removal was 50%. But under the tungsten lamp, the dye removal percentage was up to 91% for rhodamine, and 90% removal was observed for alizarin. The reason behind this trend of removal was that initially there was more active sites of nanocatalysts available and more dye was adsorbed on the adsorbate. However, with the passage of time when available sites had been occupied by dye molecules, there was no space for further dye molecules to be adsorbed so an equilibrium condition was established [35].

### 2.3. Thermodynamics

**Figure 6:**
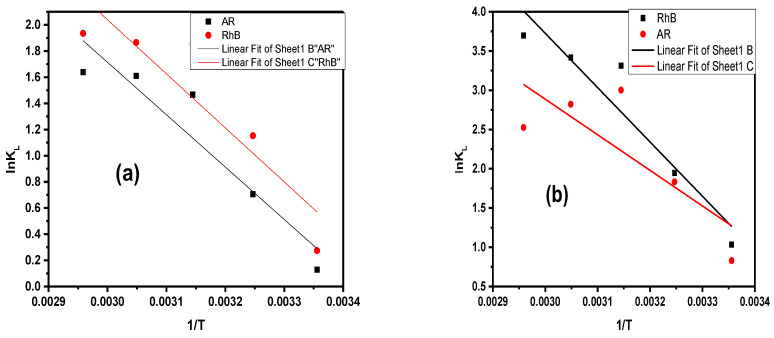
Van’t Hoff plot for RhB and AR dyes adsorption on Cu/Ni/rGO composites. (**a**) without a tungsten lamp and (**b**) with a tungsten lamp.

At variable temperatures (298, 308, 318, 328, 338), K thermodynamic parameters were examined as shown in Figure 6. Formulas that were used to study these parameters were

(2) $$lnK_{C}=\frac{∆S{}^{\circ}}{R}-\frac{∆H{}^{\circ}}{RT}\;$$

(3) $$K_{C}=\frac{C_{ad}}{C_{e}}$$

∆G° = −RTlnK_c_ (4)

Calculated values of parameters are shown in Table 2 and Table 3. In both experiments, i.e., under a tungsten lamp and without a tungsten lamp, the positive value of ∆H represents the endothermic nature of dye degradation on the nanocatalyst. The value of entropy change (∆S) was also positive which indicated good affinity of dyes on the adsorbate. Negative value of Gibbs free energy confirmed the spontaneity of dye adsorption. The value of ∆G increases with increasing temperature that represented the feasibility of reaction [36].

Comparison of Cu/Ni/rGO with other adsorbing material is shown in Table 4.

### 2.4. Adsorption Isotherms

**Figure 7:**
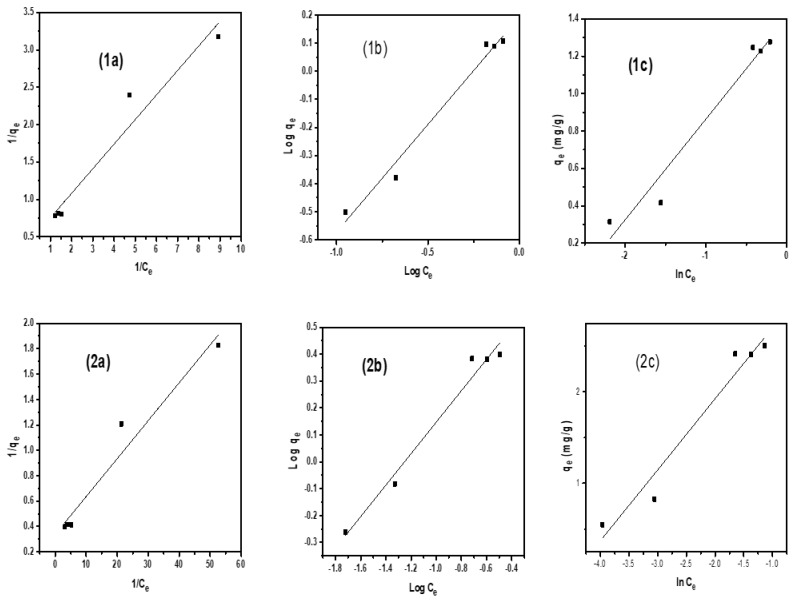
Isotherms for Rhodamine B dye (**1**) without a tungsten lamp; (**1a**) Langmuir, (**1b**) Freundlich, (**1c**) Temkin (**2**) with a tungsten lamp; (**2a**) Langmuir (**2b**), Freundlich (**2c**) Temkin.

**Figure 8:**
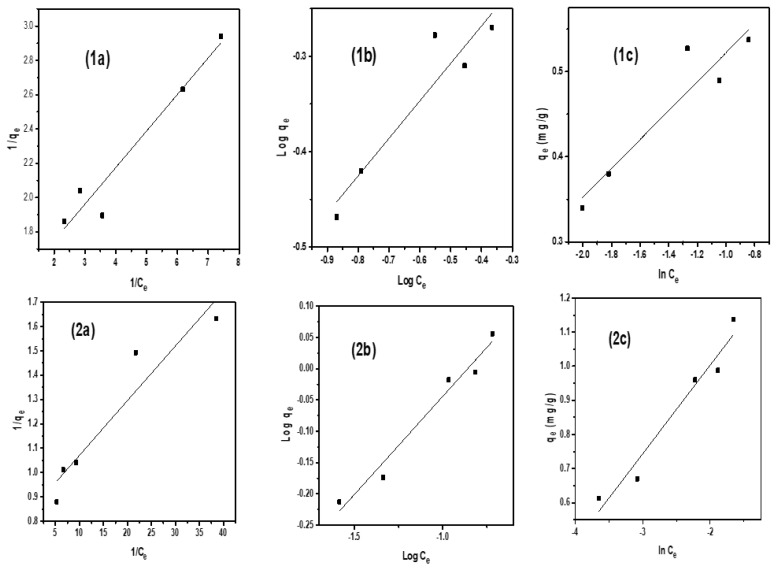
Isotherms for alizarine red dye (**1**) without a tungsten lamp; (**1a**) Langmuir (**1b**), Freundlich (**1c**), Temkin (**2**) with a tungsten lamp; (**2a**) Langmuir, (**2b**) Freundlich, (**2c**) Temkin.

To elaborate the interaction of dye molecules with the nanocatalyst, three adsorption isotherms were applied to the experimental data: Langmuir, Freundlich, and Temkin isotherms as shown in Figure 7 and Figure 8. These isotherms provide information about adsorption capacity of a synthesized composite and behavior of dye molecules on the catalyst surface [42]. Calculated values of all these isotherms are represented in Table 5 and Table 6.

For the Langmuir isotherm, the following equation was used

(5) $$\frac{1}{q_{e}}=\frac{1}{K_{L}q_{m}}\times \frac{1}{C_{e}}+\frac{1}{q_{m}}$$

According to the Langmuir isotherm, the adsorbate surface is homogeneous and monolayer adsorption occurs on it. During both experimental conditions, i.e., without a tungsten lamp and under a tungsten lamp, it can be seen that the value of R^2^ is near unity for both dyes. In order to find if either adsorption process is favorable or unfavorable, the separation factor (R_L_) is used and calculated by the following equation:

(6) $$RL=\frac{1}{1+KlCi}$$

where Kl is the Langmuir constant (L/mg) and Ci is initial concentration (mg/L).

RL = 0 is irreversible, RL = 1 is linear, RL > 1 is unfavorable, and 0 < RL < 1 is favorable, The value of RL for both dyes, i.e., rhodamin and alizarin, is less than 1, which indicated that adsorption of both dyes are favorable.

The Freundlich isotherm was also applied to analyze the heterogeneous distribution on an adsorbent surface. The following equation was used

(7) $$\log q_{e}=logK_{f}+\frac{1}{n}\log C_{e}$$

Here n represents the heterogeneity factor and is also employed to check the linearity of adsorption. If the value of n = 1, it confirms that adsorption is linear, while n > 1 gives information about the chemical nature of the adsorption process, and n < 1 represents the physical nature. In both experimental conditions for both dyes, the value of n is less than 1 confirms the physical nature of the adsorption process. The value of the correlation coefficient R^2^ is higher than the Langmuir isotherm value for both dyes and is a better fit of experimental data than the other isotherm equation.

The Temkin isotherm provides information that during the sorption process, the free energy of dye molecules decreases linearly. The following equation for the Temkin adsorption isotherm was used

(8) $$q_{e}=\frac{RT}{B\;ln(k_{T})}+\frac{RT}{B\;ln(C_{e})}$$

where q_e_ is amount of adsorbate adsorbed at equilibrium, T is temperature (K), B is constant associated to heat of sorption, R is gas constant. Values of constants were calculated from the plot Figure 8(2c) and given in Table 6 and Table 7 for both with and without a tungsten lamp.

### 2.5. Adsorption Kinetics

To obtain information about the rate of reaction and rate constants, kinetic studies were carried out. It is also important to investigate the time factor on the adsorption capacity of synthesized material. The following mathematical formula was used for the Pseudo 1st order model;

(9) $$\ln\left(q_{e}-q_{t}\right)=lnq_{e}-k_{1}t$$

The Pseudo 2nd order model was applied with following equation;

(10) $$\frac{t}{q_{t}}=\frac{1}{k_{2}q_{e^{2}}}+\frac{t}{q_{e}}$$

**Figure 9:**
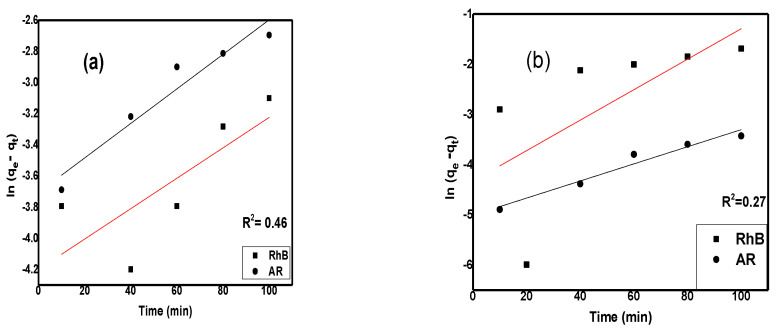

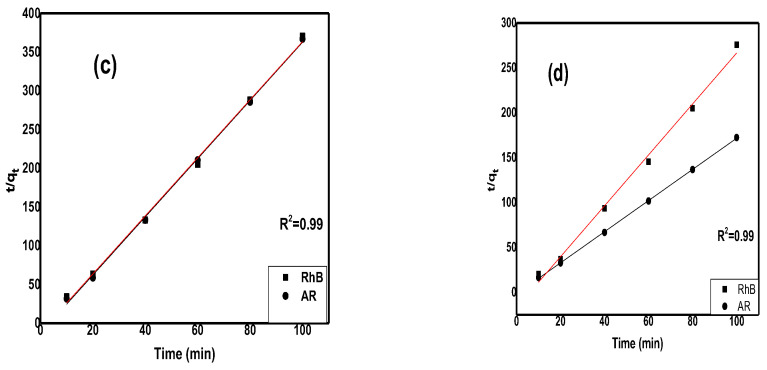
Pseudo 1st order for rhodamine B and alizarin R dye (**a**) without a tungsten lamp, (**b**) with a tungsten lamp and Pseudo 2nd order (**c**) without a tungsten lamp, (**d**) with a tungsten lamp.

Here, k_2_ (min^−1^) represents the rate constant of Pseudo 2nd order, and the graph was plotted between t/q_e_ versus time t (Figure 9c,d).

Calculated values from intercept and slope of straight-line graphs are shown in Table 6. The value of R^2^ for both dyes is very close to 1 for pseudo 2nd order. Therefore, it is concluded that, in both cases, adsorption of dyes on composites followed the pseudo 2nd order reactions [43].

### 2.6. Ionic Interferences

**Figure 10:**
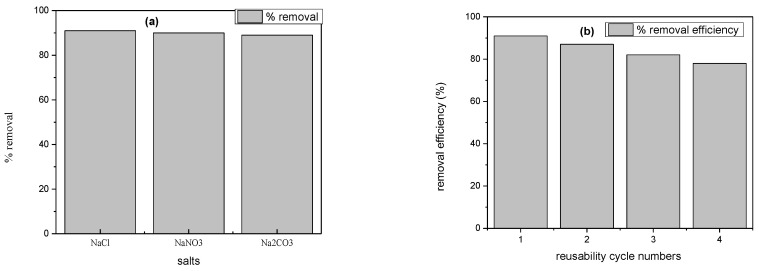

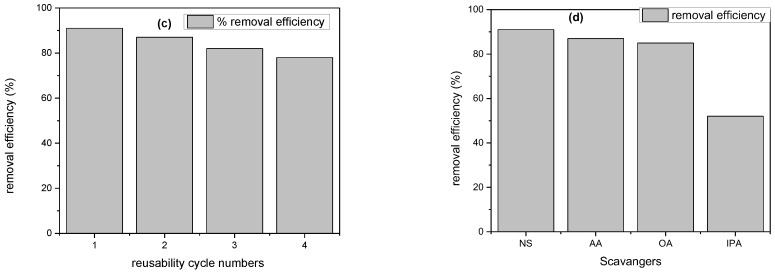
(**a**) Interferences of ions on adsorption capacity of Cu/Ni/rGO composites, Reusability of Cu/Ni/rGO composites (**b**) rhodamine, (**c**) alizarin radical, and (**d**) scavenging experimental results plot.

In industries, different types of additives like salts and surfactants are utilized. These additives can enhance or depress the interaction of dye molecules with nanocomposites. Therefore, it is essential to consider the impact of electrolytes in the degradation study [44]. To investigate the interaction of a catalyst with dye molecules, 0.1 M salt solutions of NaCl, NaNO_3,_ and Na_2_CO_3_ were prepared. A fixed amount of adsorbent, i.e., 0.02 g, was added into three beakers (100 mL) containing prepared salt solutions and dye solutions (25 mL). The reaction mixture was placed on an orbital shaker for about 1 h. After examining the results, it was concluded that the presence of these interfering ions has not any marked differences on the adsorption capacity of synthesized Cu/Ni/rGO composites Figure 10a.

### 2.7. Recyclability of Cu/Ni/rGO Nanocomposites

Regeneration studies play an important role in understanding the mechanism of degradation. As in the degradation experiments, acidic pH (4) and alkaline pH (10) are important for the maximum interaction of cationic and anionic dyes on the synthesized composite. Therefore, both acidic and basic solutions were used for the desorption of dyes from the adsorbent. For desorption of adsorbed cationic dye, rhodamine 0.01 M HCl solution was used, and 0.01 M NaOH solution was used for desorption of anionic dye, i.e., alizarin. In the 1st cycle, 95% to 98% of rhodamine and alizarin dyes were recovered from the adsorbent. The loss of adsorption capacity of synthesized Cu/Ni/rGO nanocomposites was 2–3% for both dyes. It was concluded that Cu/Ni/rGO nanocomposites can be re utilized about four times, as shown in Figure 10b,c [45].

### 2.8. Effect of Scavengers on Photocatalytic Degradation Mechanism

To investigate the effect of the reactive specie involved in the degradation of dyes by Cu/Ni/rGO composite, three scavengers were used. Hydroxyl radicals, superoxide, and holes scavengers were added to the photocatalytic degradation experiments by taking isopropyl alcohol (IPA), ascorbic (AA) acid, and oxalic acid (OA), respectively. The % removal efficiency of dyes without adding any scavengers and with three scavengers were performed and compared, and results are shown in Figure 10d. It was concluded that 91% removal efficiency was observed without any scavenger for both dyes, while 87% and 85%removal efficiency was observed with AA and OA. It means that both scavengers’ ascorbic acid and oxalic acid play very little role in the photocatalytic mechanism. When the experiment was performed with an IPA scavenger, a very clear reduction with an efficiency of 52% was observed. Hence it was concluded that hydroxyl radicals were the main active specie involved in the photocatalytic degradation experiments [46].

## 3. Experimental

### 3.1. Chemicals

Copper chloride (CuCl_2_), nickel chloride (NiCl_2_), potassium permanganate (KMnO_4_), graphite powder, hydrogen peroxide (H_2_O_2_), sulfuric acid (H_2_SO_4_), alizarin dye, and rhodamine B dye were of analytical grade and purchased from Sigma Aldrich Chemical Company, Burlington, MA, USA.

### 3.2. Preparation of Leaf Extract

Initially plant leaves were washed and shade dried. Then, these were crushed to a fine powder. We placed 3 g of leaf powder in distilled water (100 mL) and boiled for 45 min at 70 °C. Aqueous extract was filtered with Whatman filter paper. Collected filtrate was stored at room temperature for further experiments [44].

### 3.3. Fabrication of Graphene Oxide (GO)

GO was synthesized from graphite powder by a modified Hummer’s method. We added 1 g of graphite powder to 25 mL conc. H_2_SO_4_ in a 500 mL beaker under vigorous stirring in an ice bath. Then, 3 g KMnO_4_ was added into the reaction mixture. After 3 h, 50 mL of distilled water was added drop wise, and temperature was maintained ≤50 °C. Next, 100 mL of distilled water was poured instantly into the reaction medium. In order to stop the reaction, 5 mL of H_2_O_2_ was added to precipitate unreacted MnO_4_^−^ ions into MnO_2_. Excess acid was removed from the resulting mixture by repetitive washing with distilled water and centrifuged to obtain residual product [47].

### 3.4. Preparation of Cu/Ni/rGO Composites

In order to prepare Cu/Ni/rGO composite, 50 mL of plant extract was added to 1.0 g of GO under constant stirring for 10 min. Then, 20 mL of CuCl_2_ (0.08 M) and NiCl_2_ (0.05 M) solution was added to the above reaction. The reaction mixture was kept for 8 h at 80 °C under vigorous stirring. The obtained mixture was centrifuged at 3000 rpm, and as a result, Cu/Ni/rGO composite settled down and then washed several times with distilled water. After washing, consistent material was shifted into a china dish and oven dried at 60 °C for about 24 h.

### 3.5. Characterization Techniques

SEM with EDX analysis was performed at a scale level of 5 µm, 2 µm, 1 µm, and 500 nm at a magnification of 5000×, 10,000×, 25,000×, and 50,000×. UV-vis analysis (Specord Plus 200, Analytica Jena, Jena, Germany) was performed at range of 180 nm to 320 nm. FTIR spectra of GO and nanocomposite was recorded from 4000 cm^−1^ to 500 cm^−1^ using a Nicolet 6700 FTIR spectrophotometer (Thermo Fischer Scientific, Waltham, MA, USA). Crystalline nature of prepared material was assessed by XRD spectrum analysis using an X-ray powder diffractometer (Malvern Panalytical, Malvern, UK) in the range of 0–90° with CuK_α_ radiation having 0.15406 nm wavelength.

### 3.6. Dyes Removal Studies

In order to perform batch dye removal experiments, two dyes, i.e., rhodamine and alizarine were considered. We prepared 1000 mg/L of stock solution for each dye. Different concentrations, i.e., 5 mg/L, 25 mg/L, 75 mg/L, and 100 mg/L were prepared from the stock solution. Equal concentrations and volumes of both dye solutions were mixed to form a binary system. Prepared binary solution of dyes was further used in the whole experimental work. In order to analyze the maximum dye removal capacity of synthesized nanocomposite, different factors were optimized.

### 3.7. Point of Zero Charge

To calculate point of zero charge (pzc) 8.49 g of NaNO_3_ was dissolved in 1000 mL of H_2_O. Initial pH value of prepared (0.1 M) NaNO_3_ solution was adjusted separately in the range of 2 to 12 using 0.1 M hydrochloric acid or 0.1 M sodium hydroxide solution. We added 10 mg of adsorbent to each system, and each system was placed on an orbital shaker for about 24 h. After 24 h, the final pH (pH_f_) was calculated. In order to find out the point of zero charge, a straight-line graph was plotted between pH_i_ and ∆pH (pH_i_ − pH_f_) [48].

## 4. Conclusions

The adsorbing material Cu/Ni/rGO nanocomposite was successfully synthesized by a green route. The phyto constituents from an aqueous extract of *Dypsis lutescens* were considered helpful to reduce and stabilize the synthesizing nanocomposites. This plant has never been utilized before this composites formation, and the synthesized composite was used as an adsorbent to remove more than one dye in a single step. UV-vis, FTIR, XRD, EDX, and SEM characterization analysis were performed to examine the Cu/Ni/rGO composites. The synthesized nanocomposites as an adsorbent material showed excellent removal efficiency of rhodamine B and alizarine R dye from a mixed solution of these dyes. Different factors like contact time, dye concentration, amount of adsorbent, effect of temperature, and pH factor were observed, and optimal conditions for maximum removal of dyes were concluded. Thermodynamics parameters revealed the spontaneity of reaction, endothermic nature, and good affinity of dye molecules with the adsorbent. Kinetic studies proved that the reaction of dye molecules with adsorbent followed Pseudo 2nd order. Various isotherms were used, and it was observed that the Freundlich isotherm was the best fit for both dyes’ adsorption.

## Figures and Tables

**Table 1:** Lattice parameters of Cu/Ni/rGO.

Lattice Parameters	
a (Å):	2.8839
b (Å):	2.8839
c (Å):	2.8839
Alpha (°):	90.0000
Beta (°):	90.0000
Gamma (°):	90.0000
Calculated density (g/cm^3^)	7.20
Volume of cell (10^6^ pm^3^)	23.99

**Table 2:** Thermodynamics parameter without the tungsten lamp.

Adsorbing Material	Dye	Temp (K)	K_L_	∆H° (KJ mol^−1^)	∆S° (J mol^−1^K^−1^)	∆G° (KJ mol^−1^)	R^2^
Cu/Ni/rGO composite	Rhodamine B	298	1.31	34.35	120.03	−0.67	0.77
		308	3.16			−2.95	
		318	6.36			−4.89	
		328	6.45			−5.08	
		338	6.92			−5.43	
Cu/Ni/rGO composite	Alizarin Red	298	1.13	33.29	114.13	−0.31	0.84
		308	2.02			−1.80	
		318	4.33			−3.87	
		328	5			−4.38	
		338	5.14			−4.60	

**Table 3:** Thermodynamic parameters under a tungsten lamp.

Adsorbing Material	Dye	Temp (K)	K_L_	∆H° (KJ mol^−1^)	∆S° (J mol^−1^K^−1^)	∆G° (KJ mol^−1^)	R^2^
Cu/Ni/rGO composite	Rhodamine B	298	2.81	57.54	203.60	−2.56	0.87
		308	7			−4.98	
		318	27.5			−8.76	
		328	30.45			−9.31	
		338	40.35			−10.39	
Cu/Ni/rGO composite	Alizarine Red	298	2.29	37.71	137.13	−2.05	0.52
		308	6.25			−4.69	
		318	20.11			−7.93	
		328	16.81			−7.69	
		338	12.5			−7.09	

**Table 4:** Comparison of dyes removal efficiency of Cu/Ni/rGO with Cu/Ni nanoparticles.

Adsorbing Catalyst	Synthesis Methdology	Light Source	Dye	Removal Efficiency	Degradation Time	Reference
Cu/NiO nanoparticles	Coprecipitaion method	Visible	Methylene blue	89%	50 min	[37]
Cu/NiO nanopartciles	Coprecipitaion method	Visible	Alizarin R	90%	60 min	[38]
Cu/NiO nanoparticles	Coprecipitaion method	Visible	Erichrome black-T Methylene blue	51%	90 min	[39]
Cu/NiO nanoparticles	Green synthesis (*Okra plant*)	UV-light	Methylene blue	78%	105 min	[40]
Cu/Ni nanoparticles	Green systhesis *(Zingiber officinale)*	UV-light	Crystal violet	95%	160 min	[41]
Cu/Ni/rGO	Green synthesis (*Dypsislutescens plant)*	Tungsten lamp	Rhodamin B	91%	20 min	Present work
Cu/Ni/rGO	Green synthesis (*Dypsislutescens plant)*	Tungsten lamp	Alizarin R	90%	20 min	Present work

**Table 5:** Calculated values of constants of adsorption isotherms without a tungsten lamp.

Dyes		Constants	Isotherms
Rhodamin	Alizarin		Langmuir
0.42	1.32	Intercept	
0.32	0.21	Slope	
2.35	0.75	K_L_ (L/g)	
7.16	6.20	q_m_ (mg/g)	
0.38	0.75	R_L_	
0.94	0.86	R^2^	
0.19	−0.11	Intercept	Freundlich
0.76	0.39	Slope	
0.76	0.39	1/n	
1.56	0.77	K_f_	
0.97	0.92	R^2^	
1.40	0.69	Intercept	Temkin
0.53	0.16	Slope	
0.53	0.16	BT (J mol^−1^)	
13.49	58.81	Kr (L mg^−1^)	
0.95	0.85	R^2^	

**Table 6:** Calculated values of constants of adsorption isotherms under a tungsten lamp.

Dyes		Constants	Isotherms
Rhodamin	Alizarin		Langmuir
0.33	0.84	Intercept	
0.02	0.02	Slope	
2.95	1.18	K_L_ (L/g)	
99.04	37.74	q_m_ (mg/g)	
0.34	0.65	R_L_	
0.94	0.88	R^2^	
0.73	0.26	Intercept	Freundlich
0.58	0.31	Slope	
0.58	0.31	1/n	
5.39	1.86	K_f_	
0.97	0.96	R^2^	
3.47	1.52	Intercept	Temkin
0.77	0.26	Slope	
0.77	0.26	BT (J mol^−1^)	
88.05	349.70	Kr (L mg^−1^)	
0.94	0.94	R^2^	

**Table 7:** Calculated parameters of kinetic adsorption of RhB and AR dyes on Cu/Ni/rGO composite.

		Without a Tungsten Lamp		With a Tungsten Lamp	
Kinetics Models	Constants Values	Dyes		Dyes	
		RhB	AR	RhB	AR
Pseudo 1st order	R^2^	0.46	0.91	0.27	0.94
	q_e_	0.01	0.024	0.013	0.006
	K_1_ (min^−1^)	0.00009	0.0001	0.0003	0.0001
Pseudo 2nd order	R^2^	0.996	0.998	0.993	0.999
	q_e_	0.07	0.26	0.12	0.57
	K_2_ (min^−1^)	0.006	0.005	0.007	0.178

## Data Availability

Not applicable.
